# Some international perspectives on legislation for the management of human-induced safety risks

**DOI:** 10.4102/jamba.v8i2.170

**Published:** 2016-01-13

**Authors:** Alfonso Niemand, Andries J. Jordaan, Hendrik Minnaar

**Affiliations:** 1 Disaster Management Training and Education Centre for Africa, University of the Free State, South Africa; 2 Bureau for International Risk Assessments, Helderkruin, South Africa

## Abstract

Legislation that governs the health and safety of communities near major-hazard installations in South Africa is largely based on existing legislation that had been developed in the United Kingdom and other European Union countries. The latter was developed as a consequence of several major human-induced technological disasters in Europe. The history of the evolution of health-and-safety legislation for the protection of vulnerable communities in European Union (EU) countries, France, Malaysia and the USA is explored through a literature survey. A concise comparison is drawn between EU countries, the USA and South Africa to obtain an exploratory view of whether current South-African legislation represents an optimum model for the protection of the health-and-safety of workers and communities near major-hazard installations. The authors come to the conclusion that South-African legislation needs revision as was done in the UK in 2011. Specific areas in the legislation that need revision are an overlap between occupational health and safety and environmental legislation, appropriate land-use planning for the protection of communities near major-hazard installations, the inclusion of vulnerability studies and the refinement of appropriate decision-making instruments such as risk assessment. This article is the first in a series that forms part of a broader study aimed at the development of an optimised model for the regulatory management of human-induced health and safety risks associated with hazardous installations in South Africa.

## Introduction

The society in which we live becomes more complex every day as a result of a multitude of factors such as economic development, wars, terrorist attacks, technological innovation, societal demands for wealth creation and an increased awareness of the impact of human activities on the health and safety of people (Perrow 1999). Human populations are rapidly growing to extremes where the sustainable utilisation of natural and man-made resources is stretched to the limit. Clarke (2006) summarises this situation as follows:

People are worried, now, about terror and catastrophe in ways that a short time ago would have seemed merely fantastic. Not to say that horror and fear suffuse the culture, but they are in the ascendant. And for good reason. There are possibilities for accident and attack, disease and disaster that would make September 11 seem like a mosquito bite. (p. 2)

As one of the consequences of human-induced technological disasters, and focusing on it in this article, the regulation of major-hazard installations near densely populated areas has become more critical in order to limit the human-induced safety risks to which communities are exposed.

A ‘*major-hazard installation*’ is defined in the *South African Occupational Health and Safety Act* (Act 85 of 1993) (LexisNexis Butterworths 2003a) as:

An installation(a) Where more than the prescribed quantity of any substance is or may be kept, whether permanently or temporary;(b) Where any substance is produced, processed, used, handled or stored in such a form and quantity that it has the potential to cause a major incident. (Section 1, Definitions)

In the same Act, a ‘*major incident*’ is defined Act as ‘[*a*]n occurrence of catastrophic proportions, resulting from the use of plant or machinery, or from activities at a work place’.

The awareness around environmental conservation has grown substantially around the world during the past two decades, most notably in South Africa. However, it would appear that the safety of human communities, as far as the human-induced impact of major-hazard industrial installations are concerned, has not enjoyed the same priority as environmental issues in South Africa. In its State of the Environment Report, the Department of Environmental Affairs and Tourism (2006) confirms that their focus was on the condition of the environment and natural resources (the ecological system) in South Africa. The safety impact that major-hazard installations could have on people was not addressed in the publication. One reason for this is that environmental matters in South Africa are governed by the *National Environmental Management Act* (NEMA), (Act 107 of 1998), and the Environmental Impact Assessment Regulations of August 2010 under the national and provincial Departments of Environmental Affairs (South African Government Printer 1998) whilst the Major Hazard Installation Regulations are governed by the *Occupational Health and Safety Act* (Act 85 of 1993) (LexisNexis Butterworths 2003b) under the Department of Labour. In South Africa, the prime focus of the latter is the safety and health of workers in organisations (the labour force) and to a lesser extent that of the general public.

## Background of the study

This article is the first in a series that forms part of a current broader study aimed at the development of an optimised model for the regulatory management of human-induced health and safety risks associated with major-hazard installations in South Africa. The proposed study aims to provide answers to the following key questions:

- Does the existing regulatory framework for major-hazard installations in South Africa adequately address the safety of the public around such installations through appropriate land-use planning criteria, taking into consideration that current legislation falls within the domain of the *Occupational Health and Safety Act* with its prime focus on employees?
- Under which legislation does the governance of major-hazard installations fall in other countries in the world? Is it classified as environmental or as labour-related legislation? Should the governance of hazardous installations not be consolidated into one set of legislation, governed by a centralised body?
- What lessons can South Africa learn from the experience of other countries with regard to a regulatory framework for major-hazard installations?
- What factors need to be taken into consideration when an optimised regulatory model for major-hazard installations in South Africa is developed in the interest of employers, employees and the public?

## Research methodology

The methodology followed in this paper is based on a dualistic approach:

- Research of the literature was done with specific focus on regulatory regimes for major-hazard installations in South Africa and some overseas countries, how they developed and how they compare with those of South Africa today.
- Discussions were held with key stakeholders in government and industry in South Africa, Namibia and Europe.

## Regulatory management in South Africa

Apart from the influences that major disasters had on legislation around the world, we could not find information in the literature on the development of legislative models for the management of health and safety related to major human-induced hazards.

The major industrial disaster that took place in Seveso, Italy, in 1978 shaped industrial safety regulation around the world (Homberger *et al*. 1979). In 2001, South Africa adopted the Major Hazard Installation Regulations under the *Occupational Health and Safety Act* (Act 85 of 1993). The Major Hazard Installation Regulations are based on the UK COMAH Regulations that flowed from the Seveso II Directive of the European Union. The prime focus of occupational health and safety legislation in South Africa is the safety and health of workers in organisations (the labour force) and not so much that of the general public.

South Africa is faced with the following options:

- use the latest regulations and guidelines from developed countries such as the United Kingdom to create a regulatory framework for the management of hazardous installations in industry in order to protect, firstly, the health and safety of members of the public and, secondly, workers in the industry
- integrate existing legislation that involves major-hazard installations and disasters
- ensure that an economic regime is created and maintained in local industry that is conducive to fixed investment, profitability and employment creation.

Regulatory conflict exists in South Africa in that the boundaries between environmental legislation (NEMA and Environmental Impact Assessment Regulations) and safety legislation (*Occupational Health and Safety Act* and Major Hazard Installation [MHI] Regulations) are not clearly defined, with the result that overlapping of authority occurs. In cases where environmental authorisation is required from the Department of Environmental Affairs for new hazardous installations, environmental-assessment practitioners erroneously pull the risk-assessment reports for major-hazard installations, compiled under the MHI Regulations for the Department of Labour, into the process of assessing any impact on the environment. As a result, the Department of Environmental Affairs mistakenly gained a mandate to authorise risk-assessment reports which actually resort under the Department of Labour. This causes the stipulations of the MHI Regulations to be violated, for example, public-consultation protocols.

Campbell (2013) concludes that, if South Africa is to move to a better regulation of major-hazard installations, a number of ingredients are needed:

- good regulations, backed by clear unambiguous guidance such as risk assessment
- a focus on high-hazard industries
- competent, appropriately staffed organisations
- a bigger, more competent Department of Labour
- an integrated approach to land-use planning, driven by local planning authorities.

Campbell (2013) further formulates three dimensions for comparing the regulatory management frameworks of South Africa, the USA and the European Union:

- the identification of major-hazard installations
- control over major-hazard installations
- control of land development in the vicinity of major-hazard installations.

## Regulatory management in the United Kingdom

It is important to consider the work done in the UK regarding the development of regulations for major-hazard installations.

The history of health-and-safety regulation in the UK dates back to 1833 when the first factory inspectors were appointed under the *Factories Act* of 1833. Inspectors were appointed to focus on injuries and to control the work hours of children in the textile industry. In July 1974, the Health and Safety Commission was formed under the *Health and Safety at Work Act* of 1974. Its responsibilities included the health and safety of people at work, protecting the public generally against health and safety risks, giving advice to local authorities on the enforcement of the Act and assisting persons with duties under the Act. The promotion of ongoing research and the provision of information also formed part of its responsibilities.

The Health and Safety Executive (HSE) was established in January 1975 with the responsibilities of executing the duties of the Health and Safety Commission and enforcing health-and-safety legislation in all workplaces except those regulated by local authorities.

In 2006, the Health and Safety Commission and the Health and Safety Executive merged to form one organisation called the HSE. The HSE is the national regulatory body responsible for promoting better health and safety at work in Great Britain. However, enforcement is shared to a large extent with local authorities in accordance with the Health and Safety (Enforcing Authority) Regulations of 1998. Health-and-safety legislation in the country was shaped to a large extent by evidence gathered from the occurrence of major disasters such as the following:

- the Flixborough chemical plant explosion in 1974 (28 fatalities)
- the Golborne Colliery disaster in 1979 (10 fatalities)
- the methane explosion in the Abbeystead water-pump station in 1984 (16 fatalities)
- the Putney domestic gas explosion in 1985 (eight fatalities)
- the fire at Bradford City Football Stadium in 1985 (56 fatalities)
- the fire at King’s Cross underground railway station in 1987 (31 fatalities)
- the Clapham train collision in 1988 (35 fatalities)
- the fire and explosion on the Piper Alpha offshore oil platform in 1988 (167 fatalities)
- the Hillsborough Football Stadium spectator crush in 1989 (96 fatalities)
- the Southall East Junction train collision in 1997 (seven fatalities)
- the Ladbroke Grove train collision in 1999 (31 fatalities)
- the Morecambe Bay cockle-picker drowning in 2004 (23 fatalities)
- the explosion at the Israel Chemicals Limited (ICL) plastics factory in Glasgow in 2004 (nine fatalities)
- the explosion and fire at the Buncefield fuel depot in 2005 (more than 50 injuries).

Kevin Allars (UK Health and Safety Executive 2006) expressed concern about the level of major incidents at major-hazard installations in the UK. He commented that the Health and Safety Executive formed an inspection team to work with industry to promote sharing of information on dangerous incidents and to review preventative action plans. It follows in the wake of the Buncefield incident in the UK in 2005. The UK Health and Safety Executive (2013) places a strong emphasis on this approach of information sharing and public consultation, similar to what the NEMA prescribes in South Africa.

The Health and Safety Commission undertook a comprehensive review of the UK’s health-and-safety legislation in 1992 to determine whether existing legislation was still relevant and necessary in its then form. The review also aimed to reduce the administrative burden that legislation placed on small businesses and to examine the general approach of the Health and Safety Executive regarding enforcement of the legislation. It was found that much of the current legislation was considered too voluminous, complicated and fragmented. The review report recommended the removal of 100 sets of regulations and seven pieces of primary legislation as well as the simplification of the 340 requirements for administrative paperwork. A Simplification Plan was consequently implemented by the Health and Safety Executive to reduce the legislative burden on industry.

A follow-up review of health-and-safety legislation was commissioned by the Minister for Employment of the Department of Work and Pensions in 2011. The review was undertaken by a committee under the chairmanship of Professor Ragnar Löfstedt, Director of the King’s Centre for Risk Management at King’s College. The scope of the review was to consider the combination, simplification or reduction of around 200 statutory legislative instruments owned and enforced by HSE.

The review sought evidence from government bodies, employers’ organisations, employee organisations, professional health-and-safety bodies and academics. The report concluded that:

- self-employed bodies whose activities pose no potential health-and-safety risk to others should be exempted from such legislation
- all Approved Codes of Practice be reviewed
- the UK government work more closely with the European Union to ensure that health-and-safety legislation is risk and evidence-based
- sector-specific legislation be consolidated
- the HSE should direct all health-and-safety inspections and enforcement activities in local authorities in order to be consistent and focused on high-risk workplaces
- clarification be obtained regarding the protocols of early settlements between parties in cases of civil action initiated against employers by employees and the public
- regulatory provisions imposing strict liability on employers be reviewed and aligned with the principle of ‘reasonably practicable’.

In his review, Löfstedt (2011) made a general conclusion that is appropriate to South Africa, namely that regulatory requirements in the field of health and safety are misunderstood and applied inappropriately or inconsistently. The recommendations that flowed from the review addressed streamlining the body of regulation through consolidation, re-directing enforcement activities towards workplaces where the highest risk of injury or ill-health exist and re-balancing the civil-justice system through clarification of early settlement protocols and a review of strict liability imposed on employers.

According to the Health and Safety Executive of the UK (2013), the legislative regulation of health and safety at nuclear installations in the UK received renewed attention in 1957 following a major incident at the Windscale nuclear site. It led to the passing of the *Nuclear Installations Act* in 1959, followed by the formation of the Nuclear Installations Inspectorate (NII) within the Ministry of Power. In 1975, the Health and Safety Commission established several advisory committees, including the Advisory Committee on the Safety of Nuclear Installations. In so doing, the regulatory management of nuclear installations was taken on board by the then Health and Safety Commission, today the Health and Safety Executive. Research into the safety of nuclear installations in the UK received substantial impetus with the establishment of the Nuclear Safety Research Management Unit in 1990 when the responsibility for nuclear-safety research was transferred from the UK Department of Energy to the Health and Safety Commission.

## Regulatory management in European Union countries

The complexity of and room for confusion created by risk assessment at major-hazard installations were the study topic of Ignatowski and Rosenthal (2001). For this purpose, they developed a Chemical Accident Risk Assessment Thesaurus (CARAT) in the Organization for Economic Cooperation and Development (OECD) throughthe Working Group on Chemical Accidents. The research recognises the difficultly of communicating amongst member countries about the risk assessments of hazardous installations. They conclude that this difficulty was based largely on the fact that certain ‘terms of art’ have different meanings in different countries and cultures. Furthermore, different people and organisations use different terms of art to address the same concept. This cultural complexity is a prominent feature of South African society and may contribute to the problem of legislation for major-hazard installations. The lack of consistency in the definitions of essential terms of art creates an impediment to understanding amongst all stakeholders of the approaches and methodologies used in risk assessment at major-hazard installations. This, in turn, creates uncertainty aboutthe significance of the assessment results.

Shaluf (2007) introduces the concept of technological disasters instead of major-hazard installation disasters. This helps to emphasise the industrial, man-made nature of such disasters in order to manage themeffectively. Reference is made to some major industrial disasters in the world, namely Seveso-1978, Flixborough-1974, Bhopal-1984 and Piper Alpha-1988. Shaluf (2007) refers to the following definition of a major accident as used by the International Labour Organisation:

A major accident is an occurrence such as a major emission, fire or explosion resulting from uncontrolled developments in the course of an industrial activity, leading to a serious danger to man, immediate or delayed, inside or outside the establishment and to the environment, and involving one or more dangerous substances. (p. 115)

Note that there is no reference to the labour force *per se* although the inside and outside of the facility establishment are included.

Operators of hazardous installations, in particular those with limited resources and time constraints, often find it difficult to collect the large number of different safety-performance indicators of their plants in accordance with the approaches developed by member countries in the OECD, as outlined by Mengolini and Debarberis (2008). They propose that organisations should focus on a culture of safety amongst workers (plant operators and managers) so that major disasters can be prevented. A typical example would be to involve workers in regular focus-group discussions on safety. Our proposals can form the basis of expanded regulatory measures for major-hazard installations.

Bellamy, Geyser and Wilkenson (Loss Prevention Bulletin 2008a) find that the roleof human factors in safety in hazardous work situations is often poorly understood. In practice, human factors require an understanding of human capabilities and fallibilities so as to recognise the relationship between work demands and human capacities when considering human and system performance. The aim should be to eliminate or reduce the chance of adverse human behaviour, which can lead to harm through accidents or chronic exposure to conditions adverse to health. This particular aspect relates to the situation prevailing at some major-hazard installations in third-world countries.

Major-hazard installations are needed in every country to provide for its manufacturing, agriculture, transportation and energy needs. These installations store large quantities of hazardous substances and energy in one place such as refineries, petrochemical plants, chemical production plants, storage facilities for liquid petroleum gas and water-treatment plants. Some contemporary technical installations are so complex and so closely meshed that accidents are inherent in their design. Such systems could generate ‘normal accidents’ (Perrow 1999). Perrow makes it clear that most high-risk systems have inherent characteristics that cause the accidents that take place in them to be ‘normal’, that is, inevitable as a result of complex interdependence between the various system components. He refers to the disaster at Three Mile Island’s nuclear reactor in 1979 as a case in point. Shaluf (2008) comes to the conclusion that the impact of technological disasters is not limited to the plants only but can extend to neighbouring surroundings. The establishment of disaster criteria is useful to set benchmarks for the definition of disaster incidents and to declare the need for international assistance.

Papazoglou *et al*. (2003) state that the European Union Directive 96/82/EC for the Control of Major-accident Hazards (the Seveso-II Directive) requires that major-hazard companies implement a prevention policy for major accidents and have auditable safety-management systems. The authors propose an integrated safety-management system that links technical and managerial models to give good insight into the quality of management and its influence on the safety of a plant. The quality of management is particularly relevant when the safety of major-hazard installations is considered.

Industrial safety regulations were passed in the European Community in 1982 (Council of the European Communities 1982) called the Seveso Directive by the Council of the European Communities (Council of the European Communities 1996), which imposed more harsh industrial safety regulations. The Seveso Directive was updated and is currently referred to as the Seveso-II Directive under European Union (EU) law or the Control of Major-accident Hazards (COMAH) Regulations in the United Kingdom.

Löfstedt (2011) finds that much of the health and safety legislation that applies to businesses in the UK implements EU directives. About 63% of new health-and-safety regulations introduced between 1997 and 2009 originated in the EU, and EU directives accounted for 94% of the cost of UK health-and-safety regulations introduced between 1998 and 2009.

The UK Health and Safety Executive’s (2010, 2013) Hazardous Installations Directorate has implemented a system of specialist inspectors to inspect and assess major-hazard sites under their COMAH regulations. Bellamy *et al*. (Safety Science 2008b) explain how human factors, safety-management systems and wider organisational issues fit together. This is particularly important for hazardous installations in countries where there can be a lack of such a wider focus for various reasons, one being the lack of skilled human resources.

The dilemma of comparability of assessed risks from diverse hazards is addressed by Rushton and Carter (2009) of the UK Health and Safety Executive’s Hazardous Installations Directorate. They introduce a new concept of total risk of death as measure to assist decisions on land-use planning. The concept supports more direct comparison with other risks such as everyday work and transport risks. It illustrates the problem that regulating authorities have in evaluating and authorising major-hazard installations. Table 1 gives a comparison of major-hazard installation regulations for European Union countries and South Africa.

**TABLE 1:** Comparison of major-hazard installation regulations for European Union countries and South Africa.

Dimension	European Union Countries	South Africa
1: How are major-hazard installations identified?	Legislation applies to establishments and installations where a dangerous substance is present in quantities above a certain specified threshold. The nuclear industry is excluded from the legislation. EU countries have a clear approach to assisting industry in identifying whether or not the relevant legislation applies to a particular establishment or installation.	Definition of a major-hazard installation is ambiguous. Risk assessment is prescribed as decision-making instrument. However, the assessment methodology leaves room for varying interpretations. Classification of major-hazard installations is unclear, non-specific and interpreted differently by role players and authorities in industry. There is no differentiation between various categories of hazardous installations. Legislation can create barriers to trade due to cost impact. The nuclear industry is excluded from the legislation and is covered under separate legislation.
2: How are major-hazard installations controlled?	The quantity of dangerous substances dictates the control measures. Establishments and installations fall into two groups: lower-tier sites and top-tier sites. *Lower tier establishments*: notify the regulator; prepare a major accident prevention policy; take measures to prevent major accidents; report accidents. *Top tier establishments*: As above, but with the additional requirement to submit a safety report. The regulatory approach is balanced where low-hazard installations are not burdened with disproportionate cost and administration. High-hazard installations are regulated in proportion to their scale of risk. Legislation is reviewed often such as the Löfstedt review in UK in 2011.	Notify the authorities, perform a risk assessment and developan on-site emergency-response plan. The requirements beyond risk assessment are limited to emergency-response planning, incident reporting and risk-assessment revision. The same emergency management measures are required across all industries, for all types of hazardous-installation categories, which is onerous for small operators.
3: How is development controlled in the vicinity of major-hazard installations?	Member states are responsible for implementing policies and procedures for land-use control of new establishments, modification of existing establishments and new developments around Seveso II high-risk establishments. The requirements are met differently across different member states. The Seveso II land-use planning directives vary across Europe. The process is controlled by planning authorities who are advised by technical specialists such as the Health and Safety Executive in the UK. This gives a greater degree of assurance that major hazards are taken into consideration for land-use planning. Environmental impact is not explicitly addressed.	Local authorities have the responsibility to control developments around existing major-hazard installations. The regulatory process for major-hazard installations are in some cases detached from the land-use planning process and therefore not adequately considered in development planning. The regulations are ambiguous and therefore poorly enforced. Environmental impact as defined in environmental legislation is not addressed in the regulations.

*Source*: Campbell, D., 2013, ‘PetroSA’, presentation at Major Hazard Installation Seminar, Boksburg, South Africa, 19 February

## Regulatory management in the United States of America

The United States Environmental Protection Agency (EPA) plays a decisive role in the setting and enforcement of safety standards in the USA. In its historic overview, the USA Environmental Protection Agency (2013) reports that the US President decided in 1970 to establish an independent authoritative regulatory body to enforce environmental policy. He eventually established the US Environmental Protection Agency (EPA) for this purpose. The mission of the EPA was formulated mainly to:

- establish and enforce environmental protection standards and goals
- conduct research on the adverse effects of pollution
- gather information on pollution for the development of environmental protection programmes
- provide for grants and technical assistance to combat environmental pollution.

Campbell (2013) reports that the EPA analysed the chemical incidents between 1980 and 1990 and compared it with the Bhopal disaster in December 1984 (Union Carbide Corporation 1984). It resulted in the amendment of the USA’s *Clean Air Act* of 1990 to make provision for specific mandates to enable the USA’s Occupational Safety and Health Administration (OSHA) and the USA’s EPA to establish regulations for the protection of employees in the workplace, the public at large and the environment in general. The OSHA fulfilled its mandate by promulgating the Process Safety Management Regulation in 1992 whilst EPA promulgated the Risk Management Programme Regulation in 1996. The Process Safety Management Regulation focused on on-site health-and-safety management whilst the Risk Management Programme Regulation focused on off-site health-and-safety management. Table 2 gives a comparison of major-hazard installation regulations for United States of America and South Africa.

**TABLE 2:** Comparison of major-hazard installation regulations for United States of America and South Africa.

Dimension	United States of America	South Africa
1: How are major-hazard installations identified?	Process-safety management and risk-management planning are used as decision-making instruments. Legislation is based on threshold quantities of dangerous substances and applies to processes or installations. The USA has a clear approach to assisting industry in identifying whether or not the relevant legislation applies to them.	Definition of a major-hazard installation is ambiguous. Risk assessment is prescribed as decision-making instrument. However, the assessment methodology leaves room for varying interpretations. Classification of major-hazard installations is unclear, non-specific and interpreted differently by role players and authorities in industry. There is no differentiation between various categories of hazardous installations. Legislation can create barriers to trade due to cost impact. The nuclear industry is excluded from the legislation and is covered under separate legislation.
2: How are major-hazard installations controlled?	A process-safety management system with 14 steps is required for all hazard installations. Incorporates process-hazard analysis.	Notify the authorities, perform a risk assessment and develop an on-site emergency-response plan. The requirements beyond risk assessment are limited to emergency-response planning, incident reporting and risk-assessment revision. The same emergency management measures are required across all industries, for all types of hazardous-installation categories, which is onerous for small operators.
3: How is development controlled in the vicinity of major-hazard installations?	The Environmental Protection Agency (EPA) requires a risk-management plan. It is passed on to local and state regulators. The focus is on response rather than the proactive management of development around these installations. Regulations are weak in this regard. The focus is on prevention and recovery at site rather than on separation through planning control.	Local authorities have the responsibility to control developments around existing major-hazard installations. The regulatory process for major-hazard installations are in some cases detached from the land-use planning process and therefore not adequately considered in development planning. The regulations are ambiguous and therefore poorly enforced. Environmental impact as defined in environmental legislation is not addressed in the regulations.

*Source*: Campbell, D., 2013, ‘PetroSA’, presentation at Major Hazard Installation Seminar, Boksburg, South Africa, 19 February

## Regulatory management in France

France provides us with a clear focus on land-use planning as an important consideration for the regulation of major-hazard installations. Salvi and Gaston (2004) describe the context of hazardous establishments in France as one which entails a very complex decision process based on several criteria which are difficult to evaluate. The only explicit criteria in France are those related to the consequences of accidents that are used to define the safety distances around hazardous establishments. In their code related to the control of hazardous establishments (Code de l’Environement, Livre V, 1976, Art L512), the license to operate such a facility is subordinated to a sufficient distance between the establishment and people in the vicinity. In other words, the regulatory bodies may theoretically not license new establishments that can harm people in case of a major accident, but it is easier to apply for new installations than for existing ones. This idea appeared first in an imperial act in 1810 and then in a new law published in 1917 on hazardous and insanitary plants, but it was clearly reinforced with law number 76–633 in 1976 that became the ‘Environment Code’ in France in 2000. Note that this legislation is considered to be focused on the environment in its broadest sense rather than on the labour force. It includes the general public and is administered by the French Ministry of Environment.

## Regulatory management in Malaysia

Malaysia provides a good perspective with regard to the foundation on which its regulatory framework is based. Shaluf, Ehmadun and Sharif (2003) investigate the causes of technological (major-hazard installation) disasters in Malaysia in a fireworks factory, a petrochemical plant, a refinery and another mutual major-hazard installation. They conclude that there were seven main causes of the disasters, namely:

- social errors related to operators and managers at the installations
- technical errors such as design shortcomings and equipment failure
- organisational errors related to wrong procedures and documentation (the authors particularly focus on the link between the social and technical side of the installations through policies, regulations, rules, manuals, training and emergency plans)
- operational errors caused by the human and technical interface
- warning systems used to alert the management of the facility that dangerous operational conditions are starting to arise
- triggering events after which disaster is unavoidable, such as unsafe acts and conditions
- defence errors such as a lack of emergency-response measures.

## Conclusion

This article provided a concise overview and comparison of the regulatory frameworks for major-hazard installation in EU countries, USA, France, Malaysia and South Africa. Whilst there are marked similarities between EU countries and the USA, albeit with some apparent shortcomings with regard to land-use planning, current legislation in South Africa needs revision in order to bring it on par with those of developed countries. In particular, South-African legislation should be improved in terms of the following aspects:

- The definition of a major-hazard installation is ambiguous with the result that the classification of major-hazard installations is unclear, non-specific and interpreted differently by role players, risk assessors and authorities in the industries.
- Risk assessment is prescribed as a decision-making instrument, but the assessment methodology leaves room for varying interpretations due to the lack of a uniform assessment standard.
- There is no differentiation between various categories of hazardous installations.
- The same emergency management measures are required across all industries, for all types of hazardous-installation categories, which is onerous for small operators.
- Local authorities have the sole responsibility to control developments around existing major-hazard installations, but there are no clear regulatory guidelines or measures that can legally be enforced to prevent the establishment of vulnerable developments near existing major-hazard installations.
- Apart from risk assessment, the requirements for major-hazard installation planning are limited to on-site emergency-response planning, incident reporting and risk assessment revision. This causes the regulatory process for major-hazard installations to be detached from the land-use planning process and therefore not adequately considered in development planning.
- Vulnerability studies do not form part of major-hazard installation planning at all, and aspects such as community vulnerability, resilience and coping capacity related to the specific installation are not considered at all.
- There is a gap between the Major Hazard Installation Regulations and the *Disaster Management Act*.
- Environmental impact as defined in environmental legislation overlaps with the major-hazard installation regulations and creates conflict between the relevant state departments with regard to enforcing mandates.

## Further research

The regulation of major-hazard installations in South Africa does not consider vulnerability and sustainability science. There is scope for further research into the impact of major-hazard installations on the vulnerability, coping capacity and human-induced disaster resilience of communities. We are of the opinion that the UK, followed by EU countries, is furthest advanced with regard to the implementation of legislation to regulate major-hazard installations. However, the international comparison of South African safety legislation should be expanded to other industrialised countries such as Australia, Japan, China, Singapore, Mexico and Canada. In addition, lessons learnt from EU countries and others should be researched to identify similarities with the South-African situation.
